# Teachers’ Knowledge and Attitudes toward Sustainable Inclusive Education for Students with Emotional and Behavioral Disorders

**DOI:** 10.3390/children9121940

**Published:** 2022-12-10

**Authors:** Keetam D. F. Alkahtani

**Affiliations:** Department of Special Education, College of Education, King Saud University, Riyadh 11451, Saudi Arabia; kalkahtani@ksu.edu.sa

**Keywords:** mixed method, inclusion, inclusive education, sustainable inclusive, emotional and behavioral disorders, teachers’ attitudes, teachers’ knowledge

## Abstract

With the growing number of students diagnosed with emotional and behavioral disorders (EBDs), there is a need to understand how teachers perceive those students. A mixed-method research design was used to determine whether there was a relationship between the level of general education teachers’ knowledge of emotional and behavioral disorders (EBDs) and their attitudes toward students with EBDs. The participants in the study were 782 certified elementary regular education teachers. Quantitative data were collected using two questionnaires, the Knowledge of Emotional and Behavioral Disorders Questionnaire (KEBDQ) and the General Educators’ Attitudes toward Emotional and Behavioral Disorders Questionnaire (GEAEBDQ). Descriptive analysis and Pearson correlation coefficients were used to analyze the data. The results of the quantitative data indicated that the teachers’ responses reflected both negative attitudes toward students with EBDs and poor knowledge of EBDs. A significant correlation was found between teachers’ level of knowledge and their attitudes toward students with EBDs. Qualitative data gathered from the interviews were analyzed using the thematic analysis approach. The qualitative findings are in line with the quantitative results. Implementation of professional development training to support general education teachers to acquire knowledge of EBDs may improve teachers’ perceptions of students with EBDs.

## 1. Introduction 

With the introduction of education as a fundamental human right for every person [1] and the Sustainable Development Goals (SDGs), especially SDG 4 that aims to ensure inclusive and equitable quality education and promote lifelong learning opportunities for all [1,2,3], students with disabilities have equal right to access to education [4,5]. 

Today’s schools, increasingly, are expected to provide quality education to students and be accountable for their achievements. At the same time, as a result of the inclusive schools’ movement, teachers are given more shared responsibility for students with special educational needs. As the number of school-aged children being diagnosed with emotional and behavioral disorders (EBDs) is increasing, it is critical for general education teachers to understand the needs of these students to teach them in a positive environment within the general education setting. A key to the success of students with EBDs in the general education classroom is the teacher’s attitudes toward, and knowledge of, the student’s disability. In the following sections, the inclusive education of students with EBDs, teachers’ attitudes toward students with EBDs, and teachers’ knowledge of EBDs are presented.

### 1.1. Inclusive Education of Students with EBDs 

The educational inclusion movement was introduced internationally in the Salamanca Statement and Framework for Action on Special Needs Education [1]. Inclusive education refers to educating students of all backgrounds and classifications in the same general education classroom in the school [2,3]. However, the focus of inclusive education is to develop an education system that strives for human rights; it is not specifically for learners with a disability [4]. The definition of inclusion can vary and has no specific or universal meaning. The definition of EBDs also causes confusion. UNESCO [5] proposed EBDs as an imprecise umbrella term that includes several social–emotional disabilities (such as depression, eating disorders, mood disorders, bipolar disorders, anxiety disorders, obsessive-compulsive disorders, attention deficit hyperactive disorders, conduct disorder, and severe disruptive behavior). Students with EBDs are more likely to be educated in more restrictive, nonintegrated, specialized settings than students with other disabilities [6,7]. Evans and Lunt stated that “schools are more ready to accept children with learning or physical difficulties, rather than behavior difficulties” [8] (p. 8). The low inclusion rate of students with EBDs may be due to their social–emotional skill deficits, which can lead to social withdrawal, inappropriate interactions with peers and adults, and poor academic performance [9].

### 1.2. Teachers’ Attitudes toward Students with EBDs 

Students with EBDs, by diagnosis, are confirmed to have behavioral problems that include fearfulness, unruliness, and aggression [10,11]. These behavioral problems may negatively impact classroom management and student–teacher rapport. In addition, the inclusion of students with EBDs into the mainstream classroom environment can be inextricably intertwined with teachers’ attitudes [12]. Idol reported that teachers suggested more restrictive placements for students who exhibited behavior problems [13]. Broomhead also reported that teachers did not want to have students with EBDs in their classroom due to their challenging behaviors [14]. Studies that aimed to explore teachers’ attitudes claimed that teachers have more negative perceptions toward the inclusion of students with EBDs compared to other disability groups, as they were the most difficult to include and were rejected by their teachers [15,16,17,18,19].

de Boer and colleagues reviewed 26 empirical studies on teachers’ attitudes. The results of their study indicated that teachers held the strongest positive attitudes towards students with physical and sensory disabilities, and they held the strongest negative attitudes towards the inclusion of students with behavior problems [20]. Teachers willing to facilitate the need of students with EBDs increased when the teachers had a positive attitude towards teaching students with EBDs [21].

### 1.3. Teachers’ Knowledge of EBDs 

Teachers’ knowledge of EBDs is crucial in the early stage of detection of the disorders. Teachers are the primary source of information; therefore, they are expected to contribute to the diagnostic process stage. Teachers’ knowledge of, and preparedness to, educate students with EBDs is key to the success of inclusive education. Most previous studies have focused on teachers’ attitudes, and less attention has been paid to teachers’ knowledge. Previous studies on teachers’ knowledge have examined teachers’ knowledge of ADHD as a specific disorder [22,23,24,25,26,27,28,29,30,31,32,33,34,35]. There is a lack of research concentrating on knowledge of EBDs. Anderson and Hendrickson observed that there was a significant positive correlation between teachers’ knowledge and their use of individualized support strategies for students with EBDs [36]. Teachers lacked the necessary knowledge and preparation to educate students with EBDs effectively [37]. Shillingford and Karlin also showed that teachers had insufficient knowledge; they also suggested that when teachers had knowledge or a deeper understanding of students with EBDs, they might be become more supportive of those students in their classroom [38].

The review of the literature showed that very little research has been conducted and there is a need for more research on teachers’ knowledge and attitudes with regard to educating students with EBDs. The present study focused on both teachers’ knowledge and attitudes. The study further examined the relationships between the level of teachers’ knowledge and their attitudes toward students with EBDs. The following research questions guided the present study: (1) What knowledge and attitudes do general education teachers have about students with EBDs? (2) Do teachers’ levels of knowledge correlate with their attitudes toward students with EBDs?

## 2. Method

A mixed-method design with elements of qualitative and quantitative approaches was used to overcome the limitations of a single approach [39]. In the first phase, using questionnaires, quantitative data were collected to assess teachers’ knowledge and attitudes and to identify the relationship between teachers’ knowledge of emotional and behavioral disorders and their attitudes toward students with emotional and behavioral disorders. In the second phase, using one-to-one interviews, qualitative data were collected to explore, in-depth, teachers’ opinions, knowledge of EBDs, and attitudes toward students with EBDs. Approval was obtained by the Ethics Committee of King Saud University (KSU-HR-20-446, 20 December 2020), and consent was obtained from participating teachers prior to beginning the study.

### 2.1. Participants

All certified elementary teachers from twenty urban public schools were asked to participate. All potential participants were informed that participation in the research study was voluntary and that there was no obligation to participate or to continue participating. Participants were also informed that their consent was implicitly given if they participated in the survey. They were also informed that the survey data were confidential and they were asked not to include their name or the name of their school unless they would be willing to participate in a follow-up one-to-one interview. Questionnaires and informed consent forms were sent to eight hundred and twenty-five (825) teachers, 95% of whom (*n* = 784) agreed to take part by filling out the confidential and anonymous questionnaires. Of that number, two questionnaires were not used because they were completed by special educators instead of general educators. Five teachers who completed the survey agreed to participate in a follow-up one-to-one interview. 

The demographic characteristics of the participants are listed in Table 1. Most of the participants (475; 60.8%) were females. Half of the participants (392; 50.2%) were in their thirties, 152 (19.4%) were in their forties, 134 (17.1%) were in their twenties, and 104 (13.3%) were over fifty (50) years old. Of the 782 participants, 351 (44.9%) had between 11 and 20 years of teaching experience, 209 (26.7%) had less than 10 years of teaching experience, and 222 (28.4%) had over 20 years of teaching experience. With regard to the participants’ education, the vast majority had a bachelor’s degree (761; 97.3%), while less than three percent (21; 2.7%) had a postgraduate degree. None of the participants had taken a course on teaching students with EBDs as a requirement for their teaching degree. More than ninety percent (709; 90.7%) of the participants indicated that students with EBDs should not be educated in the regular classroom. None of the participants were extremely confident in their ability to teach student with EBDs. Additionally, the vast majority (751; 96%) of the participants had no confidence in their ability to teach student with EBDs, while only four percent (31; 4%) were moderately confident that they could effectively teach a student with an EBD. Similarly, the majority (745; 95%) of the participants reported no prior knowledge about EBDs, while only five percent (37; 5%) had some knowledge. The noticeable result was that over one third (285; 36.4%) of the participants thought that they did not need in-service training regarding EBDs, although they reported poor knowledge of, and ability to teach, students with EBDs.

### 2.2. Materials and Procedure

Quantitative data were collected, in the first phase, using two questionnaires, the Knowledge of Emotional and Behavioral Disorders Questionnaire (KEBDQ) and the General Educators’ Attitudes toward Emotional and Behavioral Disorders Questionnaire (GEAEBDQ). There were no time limits imposed for completing the questionnaires, but each questionnaire took approximately 15 min to complete.

The first questionnaire, KEBDQ, was developed by Shillingford and Karlin to evaluate knowledge based on the ability to identify the symptoms of the disorder and to recognize the appropriate strategies for handling behavior problems in the classroom [38]. The KEBDQ consisted of fifteen (15) items, each either scored right or wrong and coded one (1) or zero (0), respectively. The reliability analysis of the KEBDQ items showed an overall Cronbach’s alpha value ranging from 0.158 to 0.326 [38]. Test–retest reliability was conducted within a seven-week interval on seventy-three (73) preservice teachers at the pilot stage of this study. The test–retest reliability for the KEBDQ was (*r* = 0.85). The KEBDQ was translated into Arabic by the researcher. This translation was back-translated by bilingual psychiatrist to ensure fidelity to the original English version.

The second questionnaire, GEAEBDQ, was specifically developed for the present study to examine teachers’ attitudes toward students with EBDs. The questionnaire was modeled on previous research studies [15,40,41]. The GEAEBDQ was divided into two sections. The first section gathered data related to demographics, such as sex, age, teaching experience, and the highest degree attained. Participants were asked if their undergraduate degree required them to take a course on teaching students with EBDs and if they needed in-service training to teach students with EBDs adequately. Additionally, participants were asked to rate their current level of knowledge about EBDs. The second section of the GEAEBDQ consisted of fifteen (15) Likert scale statements, which aimed to determine perceived attitudes toward students with EBDs. To prevent a mid-point-response style threat, each statement had a four-point continuum (4 = “strongly agree”, 3 = “agree”, 2 = “disagree”, 1= “strongly disagree”). Prior to the statistical analysis of the data, negatively worded statements were recoded so that all statements were scored in the same direction. The content and structure of the GEAEBDQ were reviewed by eight experts, seven of whom were university faculty members with experience and expertise in the field of special education, and one was a measurement specialist. The agreement of these experts ranged from 90% to 100%. The GEAEBDQ was revised based on the experts’ comments and slight changes were made to the questionnaire instructions and to the wording following these reviews. The GEAEBDQ was then piloted among seventy-three (73) preservice teachers to ensure clarity of wording, understandability, and reliability. The seven-week test–retest reliability was high (*r* = 0.89). The internal consistency of the GEAEBDQ was adequate (Cronbach’s alpha coefficient = 0.87).

Qualitative data were collected using one-to-one interviews. A semi-structured interview protocol with questions was used to focus on the teachers’ perceptions of their knowledge of, and attitudes toward, students with EBDs.

### 2.3. Data Analysis 

Quantitative data obtained from the teachers’ responses on the questionnaires were coded and entered into the Statistical Package of Social Sciences (SPSS for Windows Version 26) for analysis. Data normality was checked and both skewness and kurtosis values were nominal. Data were analyzed using descriptive analysis (frequency, percentages, means, and standard deviations) and Pearson correlation coefficients.

Qualitative data gathered from the interviews were transcribed by the researcher and were reviewed later by each participant to determine the accuracy of the data. NVivo qualitative data analysis software was used to manage and analyze the data. The thematic analysis approach was used to summarize, identify, and interpret key data for describing and reporting themes found in the data [42].

## 3. Results

### 3.1. Teachers’ Attitudes

The attitudes toward students with EBDs were addressed by analyzing the descriptive statistics of the teachers’ responses on the GEAEBDQ. Table 2 presents a summary of these results. Teachers’ responses to the opinionnaire reflected a tone of negative attitudes toward students with EBDs. They highly agreed on all the negative statements. The six negative statements are discussed according to the respondents’ degree of agreement. Almost all the teachers (99%) strongly agreed or agreed that students with EBDs could be best served by special educators in self-contained settings. Ninety-seven percent of the teachers believed that students with EBDs were likely to exhibit behavior problems in a regular classroom. The vast majority (41% strongly agreed and 53% agreed) of the teachers believed that the bad behavior of students with EBDs was likely to set a bad example for students without EBDs. They also (32.4% strongly agreed and 61.6% agreed) indicated that the presence of students with EBDs in a regular classroom would require them to make significant changes to the classroom procedures. Additionally, more than ninety-two percent (65% strongly agreed and 27.2% agreed) of the teachers assumed that students with EBDs required extra attention, which would be to the detriment of the other students without EBDs. More than eighty-six percent (13% strongly agreed and 73.7% agreed) of the teachers expected that the presence of students with EBDs in a regular classroom would be harmful to students without EBDs.

The responses related to the positive statements are also discussed according to the respondents’ degree of agreement. All nine positive statements received a low degree of agreement from the teachers. A minority (0.6% strongly agreed and 19.7% agreed) of the teachers believed that the presence of students with EBDs in a regular classroom would promote their social independence. Less than fifteen percent (1.4% strongly agreed and 13.3% agreed) of the teachers thought that students with EBDs should be given every opportunity to learn and experience all they can in a regular classroom. Around twelve percent (0.5% strongly agreed and 11.6% agreed) of teachers indicated that the presence of students with EBDs in a regular classroom would likely have a positive effect on their emotional development. Only seven percent (0.9% strongly agreed and 6.2% agreed) of the teachers thought that the presence of students with EBDs in a regular classroom could be beneficial for them in learning good behavior from their peers without EBDs. Less than seven percent (0.3% strongly agreed and 6.6% agreed) of teachers expected that the presence of students with EBDs in a regular classroom would encourage them to make adequate attempts to complete their assignments. Similarly, fewer than seven percent (0.4% strongly agreed and 6.4% agreed) indicated that the presence of students with EBDs in a regular classroom would promote their academic growth. Moreover, only 6.3% agreed that the presence of students with EBDs in a regular classroom would necessitate regular-classroom teachers to develop their teaching skills. A very low number (0.3% strongly agreed and 1.8% agreed) noted that the presence of students with EBDs in a regular classroom would promote the acceptance of differences on the part of students without EBDs. Finally, less than two percent (1.7%) of the teachers supposed that the presence of students with EBDs would not make maintaining order in a regular classroom more difficult for teachers.

### 3.2. Teachers’ Knowledge of EBDs

The results of the teachers’ responses on the KEBDQ indicated that there was a lack of knowledge regarding EBDs. The results are reported in Table 3. Analysis of the participants’ total score revealed that most of the participants answered 3–6 questions correctly with a mean score of 4 (*SD* = 1.20). None of the participants answered all the questions correctly. Five participants obtained the highest score of 9 out of a possible 15 points. Participants’ responses to each question showed that question number 11, which was “Adolescents who exhibit conduct problems are only going through their puberty phase and will outgrow it by adulthood”, had the most correct answers (377; 48.2%); whereas question number 14, which was “A child who is not overactive, but fails to pay attention, may have ADHD”, had the most incorrect answers (629; 80.4%). The KEBDQ questions can be obtained from Shillingford and Karlin [38] (pp. 192–194).

### 3.3. Relationship between Teachers’ Attitudes toward and Knowledge of EBDs 

Pearson’s correlation coefficient was computed to examine the relationship between the teachers’ level of knowledge of EBDs (as measured by the KEBDQ) and their attitudes toward students with EBDs (as measured by the GEAEBDQ).

As shown in Table 4, a strong correlation (*r* = 0.83, *p* < 0.001) was found. Teachers’ level of knowledge correlated with their attitudes toward students with EBDs. This finding indicated that teachers with higher levels of knowledge of EBDs held more positive attitudes toward students with EBDs than those with lower levels of knowledge of EBDs.

### 3.4. Qualitative Results

Only five teachers agreed to participate in a follow-up one-to-one. The results of the thematic analysis of the teachers’ interviews revealed three major themes. These themes were: insufficient knowledge of EBDs, negative attitudes toward students with EBDs, and the need for training and professional development.

Theme one: insufficient knowledge. All participants reported a lack of knowledge and feelings of unpreparedness as barriers to teaching students with EBDs. One teacher stated: “I think the behavior of students with EBDs is difficult to manage and can set a bad example for other students in my class. I do not feel that I am prepared or qualified to appropriately address behavioral issues of students with EBDs”. Another teacher said: “I will feel some anxiety if I have a student with EBDs. I have a very little information about this disability, I think I’m not able to meet the needs of students with EBDs”.

Theme two: negative attitudes. Most of the participants had a negative attitude. One teacher stated: “students with EBDs cannot control their behavior, which can disrupt class activities; therefore, they should be taught in special classes”. Another teacher was positive but still expressed some concerns of the need for trained professionals: “I have a great love and respect for students with disability. My hope would be that students with EBDs would grow and learn from other students how to form friendships, but I believe these students cannot be successful in the general education classroom as they mostly need regular counselling sessions and medication”. A third teacher cited the safety of students as a concern: “My only issue would be that because students with EBDs are highly unpredictable and have violent tendencies, it should be a great deal of consideration before they placed in a general education classroom. I’m not trying to make excuses, but I take my student safety very seriously”.

Theme three: the need for training and professional development. All the participants agreed that their teacher preparation programs did not adequately prepare them for working with students with EBDs. One teacher stated: “During my undergraduate teacher preparation program, I have had zero classes on how to handle students with EBDs”. Participants stated a need for training targeting the needs of students with EBDs in the classroom. A teacher stated: “I think the greatest challenge to have a student with EBDs is that I am afraid of making matters worse for the student because I am not properly trained. It is helpful to have more professional development in meeting the needs of EBDs students”. Participants’ responses also reflected interest in having training specific to working with students with EBDs. A teacher stated, “I would like have training on a yearly basis as well as any other professional development opportunities to learn more about students with EBDs”.

## 4. Discussion

Overall, there were three convergent findings from the quantitative and qualitative results. The first finding was that most of the participants had poor knowledge of EBDs. This result was consistent with the previously published research that found that teachers consistently reported inadequate knowledge of EBDs [36,37,38]. Insufficient knowledge may relate to teachers’ professional preparation programs, as none of the participants had taken a course on teaching students diagnosed with EBDs as a requirement for their teaching degree. Interestingly, although most of the participants reported low levels of knowledge of, and the ability to teach, students diagnosed with EBDs, some of them also reported they did not need in-service training to be adequately prepared to teach and support students with EBDs in a general education setting. This might reflect the negative attitudes toward students with EBDs, as a result of the teachers’ poor knowledge of EBDs.

The second finding was that most of the participants expressed negative attitudes towards students diagnosed with EBDs. This result was also supported by previous studies. Kauffman and Badger concluded that general education teachers viewed students diagnosed with EBDs differently than their peers [43,44,45,46,47]. Students diagnosed with EBDs were viewed by teachers as the least liked and the most difficult to teach in a classroom [15,20,48,49,50,51,52,53]. Monsen and colleagues stated that when teachers hold negative attitudes toward the inclusion of students with EBDs, there is a risk that “the teachers’ negative attitudes of students with EBDs have implications for the success of the overall inclusive education” [54] (p. 125). It is clear in this study that the limited knowledge of EBDs impacted the teachers’ attitudes towards students diagnosed with EBDs. The correlation coefficients reported in this study (Table 4) also provided interesting information. The teachers’ attitudes towards students diagnosed with EBDs were found to be strongly associated with their knowledge. This result indicates that the more knowledge teachers possess about EBDs, the more likely it is that they will hold a positive perception and accept students diagnosed with EBDs in their classroom. When teachers did not fully understand the disability, more negative perceptions occurred in comparison to when they were educated about the disability [23]. Teachers should be trained to examine their feelings about students with EBDs and work on changing any negative attitudes that might tend to blame the students for their behaviors [55]. Once teachers understand their attitudes about students with EBDs, they mostly begin to develop and learn teaching strategies to meet the needs of students with EBDs [56,57].

The final result of this research pointed to the need for training and professional development for teachers regarding students with EBDs. Previous studies have shown similar results, wherein teachers often lack the preparedness and feel not trained to teach students with EBDs [37,58,59,60]. This underscores the need to prepare teachers for teaching students with EBDs in inclusive settings with typically developing peers. James Kauffman, a leading researcher on students with EBDs, who was interviewed by Kaff and colleagues, stated that teachers should be trained to support their students by providing the best instruction [44,61,62]. Teacher programs should prepare teachers to be skilled and capable of teaching students with EBDs in a general education setting [38,63,64].

## 5. Limitations

The results of this study must be interpreted in light of several limitations. The self-selected sample of teachers who were willing to take part in the study and the small size of the participants make it difficult to generalize the findings across different populations. Additionally, similar to most questionnaire-based studies, using a questionnaire to gather information has inherent limitations [39,65,66]. The self-reports by participants may not coincide with actual situations, as they may interpret questions differently, and their responses may have varied meanings. Additionally, methodological problems also contributed to the limitations, as the measure used to evaluate the attitudes toward EBDs, the GEAEBDQ, was developed by the researcher. Although efforts were made to revise and improve the GEAEBDQ prior to its use in this study, which resulted in acceptable internal consistency, further construction and evaluation of this measure will likely increase its reliability.

## 6. Conclusions

The nature of the behavior exhibited by some students with EBDs and the limited teacher knowledge of EBDs could lead to teachers’ negative attitudes toward students with EBDs. Based on the results of this study and former studies, it is strongly suggested that teachers have access to ongoing training and professional development targeting the needs of students with EBDs in inclusive classroom settings. Teachers’ attitudes were correlated positively with their level of knowledge of EBDs. Therefore, raising teachers’ knowledge and understanding of EBDs may have a major effect on the enhancement of positive attitudes toward students with EBDs. This may aid teachers to shift their focus to how to teach and support students with EBDs to have an opportunity to be successful in the least restrictive environment. 

## Figures and Tables

**Table 1:** Demographic information of the of the Participants (*n* = 782).

Descriptor	*n* (%)
Sex	
Male	307 (39.2)
Female	475 (60.8)
Age	
20–30 Years	134 (17.1)
31–40 Years	392 (50.2)
41–50 Years	152 (19.4)
51 Years and Above	104 (13.3)
Teaching Experience	
1–10 Years	209 (26.7)
11–20 Years	351(44.9)
21 Years and Above	222 (28.4)
Teacher’s Educational Level	
Bachelor’s	761 (97.3)
Master’s	21(2.7)
Doctorate	0 (0)
Did your undergraduate degree require you to take a course on teaching students with EBDs?	
Yes	0 (0)
No	782 (100)
Should students with EBDs be educated in the regular classroom?	
Yes	73 (9.3)
No	709 (90.7)
How confident are you that you could effectively teach a student with EBDs?	
Not at All Confident	751 (96)
Moderately Confident	31 (4)
Extremely Confident	0 (0)
How do you rate your current level of knowledge about EBDs?	
Not at All Knowledgeable	745 (95)
Moderately Knowledgeable	37 (5)
Extremely Knowledgeable	0 (0)
Do you think that you need in-service training to teach adequately students with EBDs in inclusive settings?	
Yes	497 (63.6)
No	285 (36.4)

**Table 2:** Participants’ responses on the GEAEBDQ (*n*= 782).

Items	Strongly Agree *n* (%)	Agree *n* (%)	Disagree *n* (%)	Strongly Disagree *n* (%)
1. Students with EBDs are likely to exhibit behavior problems in a regular classroom.	568 (72.6)	195 (25)	19 (2.4)	0 (0)
2. Students with EBDs can be best served by special educators in self-contained settings.	561 (71.70)	213 (27.30)	8 (1)	0 (0)
3. The presence of students with EBDs in a regular classroom will promote acceptance of differences on the part of students without EBDs.	2 (0.30)	14 (1.80)	452 (57.80)	314 (40.10)
4. The presence of students with EBDs in a regular classroom can be beneficial for them in learning good behavior from their peers without EBDs.	7 (0.90)	49 (6.20)	531 (67.90)	195 (25)
5. The bad behavior of the students with EBDs is likely to set a bad example for students without EBDs.	321 (41)	414 (53)	47 (6)	0 (0)
6. The presence of students with EBDs in a regular classroom will promote their academic growth.	3 (0.40)	50 (6.40)	577 (73.80)	152 (19.40)
7. The presence of students with EBDs in a regular classroom will encourage them to make adequate attempts to complete their assignments.	2 (0.30)	52 (6.60)	575 (73.50)	153 (19.60)
8. Students with EBDs require extra attention, which will be to the detriment of the other students without EBDs.	508 (65)	213 (27.20)	61 (7.80)	0 (0)
9. The presence of students with EBDs in a regular classroom will necessitate regular classroom teachers to develop their teaching skills.	0 (0)	49 (6.30)	596 (76.20)	137 (17.50)
10. The presence of students with EBDs in a regular classroom will require teachers to make significant changes in the classroom procedures.	253 (32.40)	482 (61.60)	47 (6)	0 (0)
11. The presence of students with EBDs in a regular classroom will promote their social independence.	5 (0.60)	83 (19.70)	561 (71.70)	133 (17)
12. The presence of students with EBDs will not make maintaining order in a regular classroom more difficult for teachers.	0 (0)	13 (1.70)	653 (83.50)	116 (14.80)
13. The presence of students with EBDs in a regular classroom will likely have a positive effect on their emotional development.	4 (0.50)	91 (11.60)	631 (80.70)	56 (7.20)
14. The presence of students with EBDs in a regular classroom can be harmful for students without EBDs.	102 (13)	576 (73.70)	97 (12.40)	7 (0.90)
15. Students with EBDs should be given every opportunity to learn and experience all they can in a regular classroom.	11 (1.40)	104 (13.30)	667 (85.30)	0 (0)

**Table 3:** Participants’ responses on the KEBDQ (*n*= 782).

Items	Number of Responses	
	Correct *n* (%)	Incorrect *n* (%)
1	284 (36.3)	498 (63.7)
2	287 (36.7)	495 (63.3)
3	311 (39.8)	471 (60.2)
4	297 (38)	485 (62)
5	257 (32.9)	525 (67.1)
6	235 (30)	547 (70)
7	173 (22.1)	609 (77.9)
8	214 (27.4)	568 (72.6)
9	320 (40.9)	462 (59.1)
10	271 (34.7)	511 (65.3)
11	377 (48.2)	405 (51.8)
12	197 (25.2)	585 (74.8)
13	211 (30)	571 (73)
14	153 (19.6)	629 (80.4)
15	339 (43.4)	443 (56.6)

**Table 4:** The Mean, Standard Deviation, and *r* Value of the KEBDQ and GEAEBDQ scores.

Variable	*n*	Mean	Standard Deviation	*r*	Significance (Two-Tailed)
KEBDQ	782	34.4	27.3		
GEAEBDQ	782	3.2	3.20	0.83	0.000

## Data Availability

The data presented in this study are available on request from the author.

## References

[1] UNESCO (1994). The Salamanca Statement and Framework for Action on Special Needs Education: Adopted by the World Conference on Special Needs Education, Access and Quality.

[2] Huber K.D., Rosenfeld J.G., Fiorello C.A. (2001). The differential impact of inclusion and inclusive practices on high, average, and low achieving general education students. Psychol. Sch..

[3] Valeo A. (2008). Inclusive education support systems: Teacher and administrator views. Int. J. Spec. Educ..

[4] Thomas G., Loxley A. (2001). Deconstructing Special Education and Constructing Inclusion.

[5] UNESCO (2009). Policy Guidelines on Inclusion in Education.

[6] Gidlund U. (2018). Why teachers find it difficult to include students with EBD in mainstream classes. Int. J. Incl. Educ..

[7] Simpson R.L. (2004). Inclusion of students with behavior disorders in general education settings: Research and measurement issues. Behav. Disord..

[8] Evans J., Lunt I. (2002). Inclusive education: Are there limits?. Eur. J. Spec. Needs Educ..

[9] Lane K.L., Wehby J., Barton-Arwood S.M. (2005). Students with and at risk for emotional and behavioral disorders: Meeting their social and academic needs. Prev. Sch. Fail. Altern. Educ. Child. Youth.

[10] Allday R.A., Hinkson-Lee K., Hudson T., Neilsen-Gatti S., Kleinke A., Russel C.S. (2012). Training general educators to increase behavior-specific praise: Effects on students with EBD. Behav. Disord..

[11] Davis S.D., Young E.L., Hardman S., Winters R. (2011). Screening for emotional and behavioral disorders. Princ. Leadersh..

[12] Stoutjesdijk R., Scholte E.M., Swaab H. (2012). Special needs characteristics of children with emotional and behavioral disorders that affect inclusion in regular education. J. Emot. Behav. Disord..

[13] Idol L. (2006). Toward inclusion of special education students in general education: A program evaluation of eight schools. Remedial Spec. Educ..

[14] Broomhead K.E. (2013). Preferential treatment or unwanted in mainstream schools? The perceptions of parents and teachers with regards to pupils with special educational needs and challenging behavior. Support Learn..

[15] Cassady J.M. (2011). Teachers’ attitudes toward the inclusion of students with autism and emotional behavioral disorder. Electron. J. Incl. Educ..

[16] Cook B.G. (2001). A comparison of teachers’ attitudes toward their included students with mild and severe disabilities. J. Spec. Educ..

[17] Cook B.G., Cameron D.L. (2010). Inclusive teachers’ concern and rejection toward their students: Investigating the validity of ratings and comparing student groups. Remedial Spec. Educ..

[18] Dupoux E., Wolman C., Estrada E. (2005). Teachers’ attitudes toward integration of students with disabilities in Haiti and the United States. Int. J. Disabil. Dev. Educ..

[19] Grieve A.M. (2009). Teachers’ beliefs about inappropriate behaviour: Challenging attitudes?. J. Res. Spec. Educ. Needs.

[20] De Boer A., Pijl S.J., Minnaert A. (2011). Regular primary schoolteachers’ attitudes towards inclusive education: A review of the literature. Int. J. Incl. Educ..

[21] Breeman L.D., Wubbels T., Van Lier P.A., Verhulst F.C., van der Ende J., Maras A., Hopman J.A., Tick N.T. (2015). Teacher characteristics, social classroom relationships, and children’s social, emotional, and behavioral classroom adjustment in special education. J. Sch. Psychol..

[22] Alkahtani K.D. (2013). Teachers’ knowledge and misconceptions of attention deficit/hyperactivity disorder. Psychology.

[23] Anderson D.L., Watt S.E., Noble W., Shanley D.C. (2012). Knowledge of attention deficit hyperactivity disorder (ADHD) and attitudes toward teaching children with ADHD: The role of teaching experience. Psychol. Sch..

[24] Bekle B. (2004). Knowledge and attitudes about attention-deficit hyperactivity disorder (ADHD): A comparison between practicing teachers and undergraduate education students. J. Atten. Disord..

[25] Cueli M., Areces D., Rodríguez C., Cabaleiro P., González-Castro P. (2022). Differences between Spanish students’ and teaching professionals’ knowledge of and attitudes toward ADHD—Does knowledge influence attitude?. Psychol. Sch..

[26] Dessie M., Techane M.A., Tesfaye B., Gebeyehu D.A. (2021). Elementary school teachers knowledge and attitude towards attention deficit-hyperactivity disorder in Gondar, Ethiopia: A multi-institutional study. Child Adolesc. Psychiatry Ment. Health.

[27] Jerome L., Gordon M., Hustler P. (1994). A comparison of American and Canadian teachers’ knowledge and attitudes towards attention deficit hyperactivity disorder (ADHD). Can. J. Psychiatry.

[28] Latouche A.P., Gascoigne M. (2019). In-service training for increasing teachers’ ADHD knowledge and self-efficacy. J. Atten. Disord..

[29] Moldavsky M., Sayal K. (2013). Knowledge and attitudes about attention-deficit/hyperactivity disorder (ADHD) and its treatment: The views of children, adolescents, parents, teachers and healthcare professionals. Curr. Psychiatry Rep..

[30] Mulholland S. (2016). ADHD-specific knowledge and attitudes of teachers (ASKAT): Development and validation of a new research instrument. Int. J. Educ. Res..

[31] Perold H., Louw C., Kleynhans S. (2010). Primary school teachers’ knowledge and misperceptions of attention deficit hyperactivity disorder (ADHD). S. Afr. J. Educ..

[32] Sciutto M.J., Terjesen M.D., Frank A.S. (2000). Teachers’ knowledge and misperceptions of attention-deficit/hyperactivity disorder. Psychol. Sch..

[33] Sciutto M.J., Terjesen M.D., Kučerová A., Michalová Z., Schmiedeler S., Antonopoulou K., Shaker N.Z., Lee J.Y., Alkahtani K., Drake B. (2016). Cross-national comparisons of teachers’ knowledge and misconceptions of ADHD. Int. Perspect. Psychol. Res. Pract. Consult..

[34] Vereb R.L., DiPerna J.C. (2004). Teachers’ knowledge of ADHD, treatments for ADHD, and treatment acceptability: An initial investigation. Sch. Psychol. Rev..

[35] West J., Taylor M., Houghton S., Hudyma S. (2005). A comparison of teachers’ and parents’ knowledge and beliefs about Attention-Deficit/Hyperactivity Disorder (ADHD). Sch. Psychol. Int..

[36] Anderson L.F., Hendrickson J.M. (2007). Early-career EBD teacher knowledge, ratings of competency importance, and observed use of instruction and management competencies. Educ. Treat. Child..

[37] Gable R.A., Tonelson S.W., Sheth M., Wilson C., Park K.L. (2012). Importance, usage, and preparedness to implement evidence-based practices for students with emotional disabilities: A comparison of knowledge and skills of special education and general education teachers. Educ. Treat. Child..

[38] Shillingford S., Karlin N. (2014). Preservice teachers’ self-efficacy and knowledge of emotional and behavioral disorders. Emot. Behav. Difficulties.

[39] Creswell J.W., Creswell J.D. (2017). Research Design: Qualitative, Quantitative, and Mixed Methods Approaches.

[40] Larrivee B., Cook L. (1979). Mainstreaming: A study of the variables affecting teacher attitude. J. Spec. Educ..

[41] Larrivee B. (1981). Effect of inservice training intensity on teachers’ attitudes toward mainstreaming. Except. Child..

[42] Clarke V., Braun V. (2017). Commentary: Thematic analysis. J. Posit. Psychol..

[43] Kauffman J.M., Badar J. (2013). How we might make special education for students with emotional or behavioral disorders less stigmatizing. Behav. Disord..

[44] Kaff M.S., Teagarden J.M., Zabel R.H. (2012). Understanding and teaching students with emotional behavioral disorders: A conversation with James Kauffman. Interv. Sch. Clin..

[45] Pit-ten Cate I.M., Markova M., Krischler M., Krolak-Schwerdt S. (2018). Promoting inclusive education: The role of teachers’ competence and attitudes. Insights Into Learn. Disabil..

[46] Chao C.N., Lai F.T., Ji M., Lo S.K., Sin K.F. (2018). Which inclusive teaching tasks represent the highest level of teacher efficacy in primary and secondary schools?. Teach. Teach. Educ..

[47] Baş G. (2022). Factors influencing teacher efficacy in inclusive education. Australas. J. Spec. Incl. Educ..

[48] Avramidis E., Bayliss P., Burden R. (2000). Student teachers’ attitudes towards the inclusion of children with special educational needs in the ordinary school. Teach. Teach. Educ..

[49] Avramidis E., Norwich B. (2002). Teachers’ attitudes towards integration/inclusion: A review of the literature. Eur. J. Spec. Needs Educ..

[50] Bowman I. (1986). Teacher training and the integration of handicapped pupils: Some findings from a fourteen nation UNESCO study. Eur. J. Spec. Needs Educ..

[51] Bornman J., Donohue D.K. (2013). South African teachers’ attitudes toward learners with barriers to learning: Attention-deficit and hyperactivity disorder and little or no functional speech. Int. J. Disabil. Dev. Education..

[52] Greene R.W., Beszterczey S.K., Katzenstein T., Park K., Goring J. (2002). Are students with ADHD more stressful to teach? Patterns of teacher stress in an elementary school sample. J. Emot. Behav. Disord..

[53] Scruggs T.E., Mastropieri M.A. (1996). Teacher perceptions of mainstreaming/inclusion, 1958–1995: A research synthesis. Except. Child..

[54] Monsen J.J., Ewing D.L., Kwoka M. (2014). Teachers’ attitudes towards inclusion, perceived adequacy of support and classroom learning environment. Learn. Environ. Res..

[55] Bambara L.M., Nonnemacher S., Kern L. (2009). Sustaining school-based individualized positive behavior support: Perceived barriers and enablers. J. Posit. Behav. Interv..

[56] Kern L., Hilt-Panahon A., Sokol N.G. (2009). Further examining the triangle tip: Improving support for students with emotional and behavioral needs. Psychol. Sch..

[57] Hunt T.K., Mullen C.A. (2021). Teacher strategies used to achieve desired outcomes for students with emotional disabilities. Handbook of Social Justice Interventions.

[58] George N.L., George M.P., Gersten R., Grosenick J.K. (1995). To leave or to stay? An exploratory study of teachers of students with emotional and behavioral disorders. Remedial Spec. Educ..

[59] Lopes J.A., Monteiro I., Sil V., Rutherford R.B., Quinn M.M. (2004). Teachers’ perceptions about teaching problem students in regular classrooms. Educ. Treat. Child..

[60] Benítez-Lugo M.L., Pinero-Pinto E., Leon-Larios F., Medrano-Sánchez E.M., de-la-Casa-Almeida M., Suarez-Serrano C. (2021). Inclusive design in the field of education from the paradigm of early intervention. Children.

[61] Leggio Ph D.J.C., Terras Ed D.K.L. (2019). An investigation of the qualities, knowledge, and skills of effective teachers for students with emotional/behavioral disorders: The teacher perspective. J. Spec. Educ. Apprenticesh..

[62] McCracken T., Chapman S., Piggott B. (2020). Inclusion illusion: A mixed-methods study of preservice teachers and their preparedness for inclusive schooling in Health and Physical Education. International J. Incl. Educ..

[63] O’Farrell P., Wilson C., Shiel G. (2022). Teachers’ perceptions of the barriers to assessment of mental health in schools with implications for educational policy: A systematic review. Br. J. Educ. Psychol..

[64] Mullen C.A., Hunt T.K. (2022). Emotional disability and strategies for supporting student outcomes: Interviews with K–12 special education teachers. Teach. Dev..

[65] Timans R., Wouters P., Heilbron J. (2019). Mixed methods research: What it is and what it could be. Theory Soc..

[66] Manzoor A. (2020). Designs of mixed method research. Cognitive Analytics: Concepts, Methodologies, Tools, and Applications.

